# 

**Published:** 1880-10

**Authors:** 


American Health Primers. The Skin in Health and Disease. By L. Duncan Bulkley, M.D., Attending Phyiscian for Skin and Venereal Diseases, at the New York Hospital, Out Door Department, etc. Presley Blakiston, Philadelphia. 1880.

School and Industrial Hygiene. By D. F. Lincoln, M.D., Chairman Department of Health, Social Science Association. Presley Blakiston, Philadelphia. 1880.

What to do First in Accidents or Poisoning. By Chas. W. Dueles, M.D., Surgical Registrar to the Hospital of the University of Pennsylvania. Presley Blakiston, Philadelphia. 1880.

Transactions of the Medical Association of the State of Missouri, at its 23d annual session, held at Corthage, Missouri, May 18th, 19th and 20th. 1880.

Transactions of the National Association for the Protection of the Insane. 1880.

